# Maternal *Leishmania infantum* infection status has significant impact on leishmaniasis in offspring

**DOI:** 10.1371/journal.pntd.0007058

**Published:** 2019-02-13

**Authors:** Angela J. Toepp, Carolyne Bennett, Benjamin Scott, Reid Senesac, Jacob J. Oleson, Christine A. Petersen

**Affiliations:** 1 Department of Epidemiology, College of Public Health, University of Iowa, Iowa City, Iowa, United States of America; 2 Center for Emerging Infectious Diseases, University of Iowa Research Park, Coralville, Iowa, United States of America; 3 Department of Biostatistics, College of Public Health, University of Iowa, Iowa City, Iowa, United States of America; University of Notre Dame, UNITED STATES

## Abstract

Visceral Leishmaniasis is a deadly disease caused by *Leishmania infantum*, endemic in more than 98 countries across the globe. Although the most common means of transmission is via a sand fly vector, there is growing evidence that vertical transmission may be critical for maintaining *L*. *infantum* infection within the reservoir, canine, population. Vertical transmission is also an important cause of infant morbidity and mortality particularly in sub-Saharan Africa. While vertical transmission of visceralizing species of *Leishmania* has been reported around the globe, risk factors associated with this unique means of *Leishmania* transmission have not been identified therefore interventions regarding this means of transmission have been virtually non-existent. Furthermore, the basic reproductive number, (R_0_), or number of new *L*. *infantum* infections that one infected mother or dam can cause has not been established for vertical transmission, also hampering the ability to assess the impact of this means of transmission within reservoir of human hosts. Canine Leishmaniosis (CanL) is enzootic within a U.S. hunting dog population. CanL is transmitted within this population via transplacental transmission with no reported vector transmission, despite many repeated attempts to find infected sand flies associated with these dogs and kennels. This population with predominantly, if not solely, vertical transmission of *L*. *infantum* was used to evaluate the critical risk factors for vertical transmission of *Leishmania* and establish the R_0_ of vertical *L*. *infantum* infection. Evaluation of 124 animals born to eighteen dams diagnostically positive for infection with *L*. *infantum* showed that there was a 13.84x greater chance of being positive for *L*. *infantum* within their lifetime if the mother was also positive within her lifetime (RR: 13.84, 95% CI: 3.54–54.20, p-value: <0.0001). The basic reproductive number for vertically transmitted *L*. *infantum* within this cohort was 4.12. These results underscore that there is a high risk of *L*. *infantum* infection to transmit from mother to offspring. Targeted public health interventions and control efforts that address vertical transmission of *L*. *infantum* are necessary in endemic countries to eliminate visceral leishmaniasis.

## Author summary

Canine leishmaniosis (CanL) is a deadly disease caused by *Leishmania infantum* parasite, it is found in animal populations, including people, in more than 98 countries across the globe. CanL was first identified within the US in hunting dogs 1980 and then again in 1999 when a large outbreak in a kennel in New York occurred. As the US is usually not considered a tropical country, there was much debate about how this neglected, vector borne, tropical disease had made its way into these dogs. We found that within the U.S. hunting dog population CanL is transmitted from mom to pup with no reported sand fly transmission in the population, despite multiple attempts to find infected sand flies associated with these dogs. While vertical transmission of this disease has been reported in case reports around the globe, risk factors associated with this unique means of *Leishmania* transmission are not known. Furthermore, the basic reproductive number, (R_0_), or number of new infections that one infected animal can cause has not been reported for vertical transmission of *L*. *infantum*. It is important to know the R_0_ as it helps identify how infectious a route of transmission can be and therefore how easy it might be to control this infection. A cohort of 124 dogs from 18 dams was analyzed from 1999 to 2016 for factors related to vertical transmission. Offspring from dams ever diagnostically positive for infection with *L*. *infantum* were 13.84x more likely to become positive for *L*. *infantum* themselves within their lifetime (RR: 13.84 95% CI: 3.54–54.20 p-value: <0.0001). The basic reproductive number for vertically transmitted *L*. *infantum* within this cohort was 4.12. These results underscore that an infected mom is highly likely to infect her offspring if treatment is not started to prevent transmission. There is a need for any public health prevention and control efforts to address vertical as well as vector transmission of canine leishmaniosis in endemic countries.

## Introduction

Leishmaniosis is a disease caused by the obligate intracellular protozoan parasite *Leishmania infantum* [1–3]. Visceral Leishmaniasis (VL) can also be caused by *Leishmania donovani* which causes anthroponotic human visceral leishmaniasis in many countries including areas of Asia and Africa [4, 5]. Zoonotic visceral leishmaniasis (ZVL) occurs in countries where the disease is endemic/enzootic in both human and animal populations. Within these countries the parasite is transmitted primarily via the phlebotomine sand fly [6, 7], although the role of other means of transmission, particularly vertical transmission, is not known. Dogs play an important role in the ecology and control of ZVL as they are the predominant domestic reservoir for the disease, with greater than 10% seropositivity often evident in dogs prior to emergent VL observed in people [8]. Dog ownership is a risk factor of human visceral leishmaniasis in multiple endemic countries with ZVL including Iran, Ethiopia, and Brazil [9–11]. As such, control measures in locations where ZVL is prominent include insecticide treatment or culling of dogs. Dogs remain an important model system for understanding the ecology and epidemiology of VL [12–14].

In recent years vertical, and specifically transplacental, transmission of *L*. *infantum* has been shown to be able to maintain infection within population(s) of dogs [15, 16]. Dogs in Brazil have been shown to have infected *in utero* pups [17–19]. Multiple case reports and case series have identified vertical transmission of VL as an important cause of infant morbidity and mortality [20–22]. Compared to sand fly transmitted infection [23–25], there is very little known about the risk of vertical transmission in dogs or people [16, 26–29]. Therefore, understanding the impact and risk factors associated with parasite transmission *in utero* is important for education and treatment of infected mothers and for control of *Leishmania* infection within reservoir hosts.

In the United States leishmaniosis is enzootic in hunting dogs. CanL was first identified in a dog with no travel outside of the United States in 1980, but it was not until a large outbreak in a kennel in New York in 1999 that a larger scale study was performed to understand the broad burden of disease in the U.S. hunting dog population [30, 31]. Further examination found that the primary route of transmission in this population was vertical, from dam to pup [15, 32] and not via sand fly transmission despite many studies looking for infected sand flies associated with these infected dogs [33, 34]. Despite experimental studies that indicate that vector transmission of the *Leishmania infantum* found in US hunting dogs is possible, there is no evidence that vector transmission occurs naturally from the U.S. hunting dog population [34–36]. A decade of surveillance of this hunting hound population found that the prevalence of CanL from vertical transmission was higher than expected and similar to the rates seen in countries where VL is endemic [37, 38].

The basic reproductive number, R_0_, or the number of secondary infections one infected individuals can cause within a susceptible population is an important epidemiological value for public health officials interested in control and elimination of this disease in endemic countries [39]. Previous calculations of the R_0_ for leishmaniosis have been restricted as these studies did not include vertical transmission as a potential route of transmission or lacked data to assess the true rate of transmission in a population [40–42].

This study examines both *L*. *infantum* positive and negative dams their offspring over the course of their lifetime to determine risk factors associated with vertical transmission and the corresponding crude basic reproductive number of vertical transmission. We hypothesize that the crude R_0_ of vertical transmission will be greater than one: *Leishmania* will maintain infection by infecting at least one pup from a diagnostically positive dam. Understanding the risk factors associated with vertical transmission remains an important public health concern as elimination and control programs focusing on vector control does not show 100% reduction of VL in endemic countries with zoonotic disease [43–45], and vertical transmission appears to be a major risk for maintaining disease within an area or population.

## Materials and methods

### Study design

A retrospective cohort study based on data collected regarding *Leishmania infantum* infection and exposure in U.S. hunting dogs since the 1999 outbreak [33, 34, 46] was completed. A subset of dams that were diagnostically positive and never diagnostically positive were identified. All pups from these two respective groups, ever positive or never positive, were tracked to determine their *Leishmania* diagnostic status. All historical data was collected from studies performed by Centers for Disease Control and Prevention [33, 34] and the our laboratory at Iowa State University and the University of Iowa [15, 32, 35, 47–49].

### Ethics statement

All dogs were enrolled in this retrospective study with informed consent from their caretakers and all protocols followed were approved by the University of Iowa Institution Animal Care and Use Committee (IACUC) an AAALAC accredited institution following the requirements for the US National Institutes of Health Office of Laboratory Animal Welfare Assurances which operates under the 2015 reprint of the Public Health service Policy on Humane Care and Use of Laboratory Animals, under protocol #6041721.

### Animals

An active surveillance cohort of 4 large (>50 dogs each) kennels was established and observed over a 9-year period. Our laboratory visited each of these kennels biannually for at least three years, at which point two of the kennels elected to control visceral leishmaniasis in their kennel via euthanasia. Licensed veterinarians collected 1–5 cc whole blood and serum from all dogs present at these kennels. Demographic information regarding time of pregnancy, sex and age were collected. The active surveillance cohort testing period extended from 2007 to 2017. This surveillance effort started eight years, or at least one hunting-dog life-span, after the major *L*. *infantum* outbreak in 1999 with CanL surveillance performed on these same dogs passively by the CDC as reported in [33, 34].

### *Leishmania* diagnostic status PCR

DNA was isolated from canine peripheral whole blood samples collected in heparinized or ethylenediaminetetraacetic acid (EDTA) via the QIAmp DNA Blood Mini Kit (Qiagen, Valencia, CA) per manufacturer protocol. The quality and quanitty of DNA was assessed using a NanoDrop 2000 (Thermo, Scientific, Waltham, MA). Real time quantitative polymerase chain reaction (RT-qPCR) was performed as previously described with all samples run in duplicate with positive samples determined as samples with 1 or more positive wells and negative samples with no amplication in any wells [37, 49–51]. All RT-qPCR included both positive, negative control blood spiked with 10^6^ *Leishmania infantum* parasites, and negative controls. Between 2007 and 2011 kinetoplastid primer and probe targets were used. The primer and probe sequences were as follows: F 5’-CCGCCCGCCTCAAGAC, R 5’-TGCTGAATATTGGTGGTTTTGG, (Integrated DNA Technologies, Coralville, IA) and TaqMan probe, 5’-6FAM-AGCCGCGAGGACC-MGBNFQ, were used (Applied Biosystems, Foster City, CA). From 2012 to present ribosomal primer and probe targets were utilized. The sequences were as follows: F 5’-AAGTGCTTTCCCATCGCAACT, R 5’ CGCACTAAACCCCTCCAA (Invitrogen, Life Technologies, Grand Island, NY), probe: 5’ 6FAM-CGGTTCGGTGTGTGGCGCC-MGBNFQ (Applied Biosystems, Life Technologies, Grand Island, NY). Assays were performed on ABI 7000 systems until 2016 when they were run on ABI 7900 systems (Applied Biosystems). Analysis was performed using ABI 7000 System SDS Software and ABI 7900 HT Sequence Detection Systems Version 2.4.1. (Applied Biosystems).

### Serological status

Serological status was determined via the Dual Path Platform Canine Visceral Leishmaniasis (DPP CVL) assay (Chembio Diagnostic Systems Inc., Medford, NY) or via immunoflourescent anitbody test (IFAT). The DPP CVL assay detects *Leishmania*-specific anitbodies via rK28 antigen, a *Leishmania* recombinant antigen. The assay was utilized as previously described with positives determined as dogs with a test and control line appearing at 4 minutes or less [51]. All positives or questionable samples were confirmed using the Chembio microreader system. The system detects the intensity of the control and test lines. Immunoflourescent antibody test (IFAT) was utlized on canine samples before 2015. This test was performed by the Division of Parasitic Diseases at the Centers for Disease Control and Prevention as previously described [33, 52]. Positive tests were determined as tests where immunofluorescence was reported in 50% of organisms at serum dilutions equal to or above 1/64. These tests were performed without identifying each dog (blindly) and were repeated four times at each dilution to determine positivity.

### Statistical methods

Univariate analyses were performed to determine unadjusted relative risk values for dam’s age at the time of birth, diagnostic status during the year of birth, and other variables. Pearson chi-squared test and Fisher’s exact test were used to assess categorical variables against disease status. Mann-Whitney test was used to compare dam’s age between disease states as age not normally distributed. An unpaired t-test with the Welch’s correction was utlized to compare litter size between infected and uninfected groups. For assessment purposes the dam’s diagnostic status via qPCR or serology during the same year she gave birth was utilized. Feasability restrictions, the fact the gestational period for a dog is two months, prevented the researchers from obtaining information on the dam’s diagnostic status during pregnancy.

Multivariable logistic regressions were performed to determine adjusted relative risk. Due to the fact that the dam’s diagnostic status can be determined via qPCR and serology, diagnostitc status was assessed different ways through three models. One model included the overall diagnostic status of the dam (ever diagnostic positive vs never diagnostic positive), the dam’s age at the time she gave birth (older than six years of age vs younger than or equal to six years of age), and the sex of the puppy (male vs female). To further assess the dam’s diagnostic status impact a second model was created with qPCR and serology as separate variables. A third model was created separating the dam’s serology and dam’s PCR status in the year she gave birth into two explanatory variables. P-values of less than 0.05 were determined as statistically significant. Each model was fit assuming a binomial distribution with a log link function.

Kaplan-Meier time to event analysis was performed to assess whether dam’s diagnostic status altered time to pup diagnostic positive.

Basic reproductive number was calculated using dams who were ever diagnostically positive for *Leishmania*, from which their average proportion of puppies per litter that became *Leishmania* diagnostic positive was determined. Hunting dogs are a medium size dog with average litter size in the study was between 6–7 [53]. Using the average litter size, the proportion of puppies in a litter that would become positive for *Leishmania* was determined as the basic reproductive number of vertical transmission in US hunting dogs.

For all analyses, as observation of transmission of *L*. *infantum* infection was the goal, *L*. *infantum* exposure/diagnostic result status for each dog was identified as “ever diagnostically positive” for *Leishmania* or “never diagnostically positive” for *Leishmania*. Positivity was determined as qPCR positive and/or serologically positive at any point during the dog’s lifetime.

All statistical analyses were performed using SAS 9.4 (SAS Institute, Cary, NC) and Graph Pad Prism 6 (GraphPad Software, Inc, La Jolla, CA).

## Results

### Study demographics and univarate analysis

Compared to sand fly transmsision, little is known about the risk factors of vertical transmission of *Leishmania infantum*. Understanding these risk factors and the corresponding likelihood of transmission as measured by the basic reproductive number, R_0_, provides valuable information for assessing control and elimination programs for zoonotic leishmaniosis. We hypothesized that a dam’s positive *Leishmania* diagnostic status during pregnancy would be a risk factor of *L*. *infantum* transmission. A retrospective cohort study examined the health records from 130 dogs born to eighteen dams for risk factors associated with vertical transmisison and the corresponding indiviudal level basic reproductive number calculation. Six dogs were removed from analysis due to incomplete data to use in statistical models.There were eight dams identified as *Leishmania* diagnostic positive at some point in their lifetime and ten dams were diagnostically negative throughout their lives. Most dogs were not multiparous. The average litter size was 6–7 pups (**Table 1**).

**Table 1:** Dam and litter demographics based on dam’s *Leishmania* diagnostic status.

Variable	Dam *Leishmania* dx Positive (ever) (N = 8)	Dam *Leishmania* dx Negative (N = 10)
Average age of dam at pregnancy, ±SD (Min-Max)	5.14 ± 1.83 (2–9)	4.00 ± 1.70 (2–7)
Proportion of male puppies*, %, N	48.61, 35	38.64, 17
Average litter size ±SD (Min-Max)	7.02 ± 3.24 (1–15)	6.3 ± 2.58 (2–11)
Average number of previous litters (Min-Max)	0.57 (0–2)	0 (0–0)
Proportion of puppies *Leishmania* diagnostic positive ever, %, N	62.16, 46	4.00, 2

*Data incomplete due to missing information

*Leishmania* diagnostic status determined as dam ever positive via IFAT serology, DPP CVL assay, or PCR.

Dogs that ever became diagnostically positive were born to dam’s that were slighly older in age, 5.10 years compared to 4.04 years (p-value = 0.0004) and were more likely to be born to dams who had previously had at least one litter (p-value <0.0001, RR = 3.351 95% CI:2.32–4.83). There was no significant difference between *Leishmania* diagnostic outcome in male vs. female dogs. Dogs ever diagnostically positive were more likely to be from large(r) litters. This difference have been skewed by on particulalry large litter of fifteen puppies from a dam that was diagnostically positive during her year of pregnancy at six years of age. When this litter is removed the signficance of dam age and litter size is reduced.

Additional analysis shows that dogs born to a dam that was qPCR positive for *Leishmania infantum* at the time of pregnancy had a relative risk of being diagnostically positive during their lifetime **10.46x** greater than the risk than when the dam was PCR negative at the time of pregnancy (Unadjusted RR: 10.46, 95% CI: 3.57–31.82, p-value <0.0001). The dam’s serological status during the year she gave birth was also found to increase the risk of offspring testing diagnostically positive within their lifetime. Pups born to dams seropositve during the year they gave birth were 2.69x more likely to test positive for *Leishmania* within their life (Unadjusted RR: 2.69 95% CI: 1.32–5.52, p-value 0.0054, **Table 2**).

**Table 2:** Univariate analysis of risk factors for vertical transmission from dam to pup.

Variable	Pup ever *Leishmania* dx positive (N = 48)	Pup never *Leishmania* dx positive (N = 76)	P-value	Unadjusted Relative Risk
Average age of dam at pregnancy ±SD (Min-Max)	5.10 ± 1.61 (3.00–9.00)	4.04 ± 1.45 (2.00–7.00)	**0.0004**	N/A
Proportion of male puppies*, %, N	40.43, 19	47.83, 33	0.6731	0.91
Average litter size, ±SD (Min-Max)	9.5 ± 4.02 (1–15)	7.62 ± 2.51 (2–15)	**0.0049**	N/A
Proportion dam *Leishmania* PCR positive*^+^	46.15	4.41	**<0.0001**	**10.46**
Proportion dam *Leishmania* Seropositive*^+^	34.04	12.68	**0.0054**	**2.69**
Proportion dam ever *Leishmania* dx positive	95.83	36.84	**<0.0001**	**2.601**

*Data incomplete due to missing information.

^+^Diagnostic status of dam during year gave birth

Outcome defined as **pups** diagnostic positivity for *Leishmania* via qPCR or serology throughout lifetime.

### Controling for all variables, transmission of *Leishmania* is dramatically higher from dams diagnostically positive for *Leishmania*

A series of three logistic regression models were created to determine the risk factors associated with vertical transmision of *L*. *infantum*. The models were labeled as A, B, and C. Whether the puppy became diagnostically positive within their lifetime or not was used as the outcome for these models. Model A assessed a dam’s diagnostic status as ever positive for *Leishmania* during their lifetime as an explanatory variable along with age at the time of pregnancy, and sex of the dog. When adjusting for all other explanatory variables it is found that dogs born to a dam that was ever positive for *Leishmania* have a relative risk **13.84x** greater than dogs born to a dam that was never diagnostically positive (Adjusted RR: 13.84, 95% CI: 3.54–54.20, p-value 0.0002).

### Transmission of *Leishmania* is higher in dams qPCR positive for *Leishmania*

In order to assess the impact of seropositivity/ *Leishmania* exposure vs detectable parasite infection via qPCR from the blood in transmission two additional models were created; models B and C. Model B utilized a dam’s diagnostic status during the year she gave birth (positive vs negative), age of dam during pregnancy (older than six vs younger), and sex of the puppy as explanatory variables. In this model puppies born to dams diagnostically positive via qPCR or serology during the year of pregnancy were 2.27x more likely to become positive for *Leishmania* compared to dogs born to a dam that was diagnostically negative at the time of pregnancy. Model C used the dam’s qPCR status, serostatus and age during the year of pregnancy and progenys’ sex as explanatory variables. This model allows for the assessment of how parasite infection via qPCR from the blood vs seropositivity/ *Leishmania* exposure could affect *Leishmania* transmission. Pups born to a dam that was qPCR positive for *Leishmania* during pregnancy were 3.14x more likely to become positive for *Leishmania* in their lifetime (Adjusted RR: **3.14**, 95% CI: 1.37–7.18, p-value: 0.0067, **Table 3**). Two dogs born to a dam that was never qPCR or serologically positive for *Leishmania* were found to be positive during their lifetime. One dog was identified as ever qPCR positive and one as ever serologically positive.

**Table 3:** Dam ever dx positive and qPCR positive during year of pregnancy significantly associated with *Leishmania* transmission to pups.

Model	Variable	Sample Size N = number of dams n = number of puppies	Adjusted RR of pup lifetime exposure	95% CI	p-value
A	Dam ever *Leishmania* dx positive	N = 18 n = 116	**13.84**	**3.54–54.20**	**0.0002**
B	Dam dx positive during yr. of pregnancy	N = 14 n = 100	**2.27**	**1.40–3.68**	**0.0009**
C	Dam qPCR positive during pregnancy	N = 14 n = 100	**3.14**	**1.37–7.18**	**0.0067**
C	Dam seropositive during pregnancy	N = 14 n = 100	1.07	0.46–2.55	0.8639

Multivariate logistic regression analysis for risk factors associated with *Leishmania* vertical transmission. Model A: Explanatory variables are dam’s *Leishmania* diagnostic status (ever positive vs. never positive), age of dam during pregnancy (older the 6 years old vs. 6 or younger), and sex of offspring. Model B: Explanatory variables are dam’s *Leishmania* diagnostic status during pregnancy, age of dam during pregnacy, sex of offspring. Model C: Explanatory variables are dam’s *Leishmania* qPCR serostatus and age during pregnancy, sex of offspring.

A dam’s serological status during the year of pregnancy was not statistically significantly associated with her offspring becoming diagnostically positive. This was an interesting finding as qPCR is a measure of parasite DNA within the peripheral blood. As the transplacental blood supplied each *in utero* puppy with nutrients, and apparently parasites, this may have increased the risk of the puppy becoming infected with *Leishmania* parasites.

### Offspring of dams diagnostically positive for *Leishmania* are more likely to become positive for *Leishmania*

**Fig 1:**
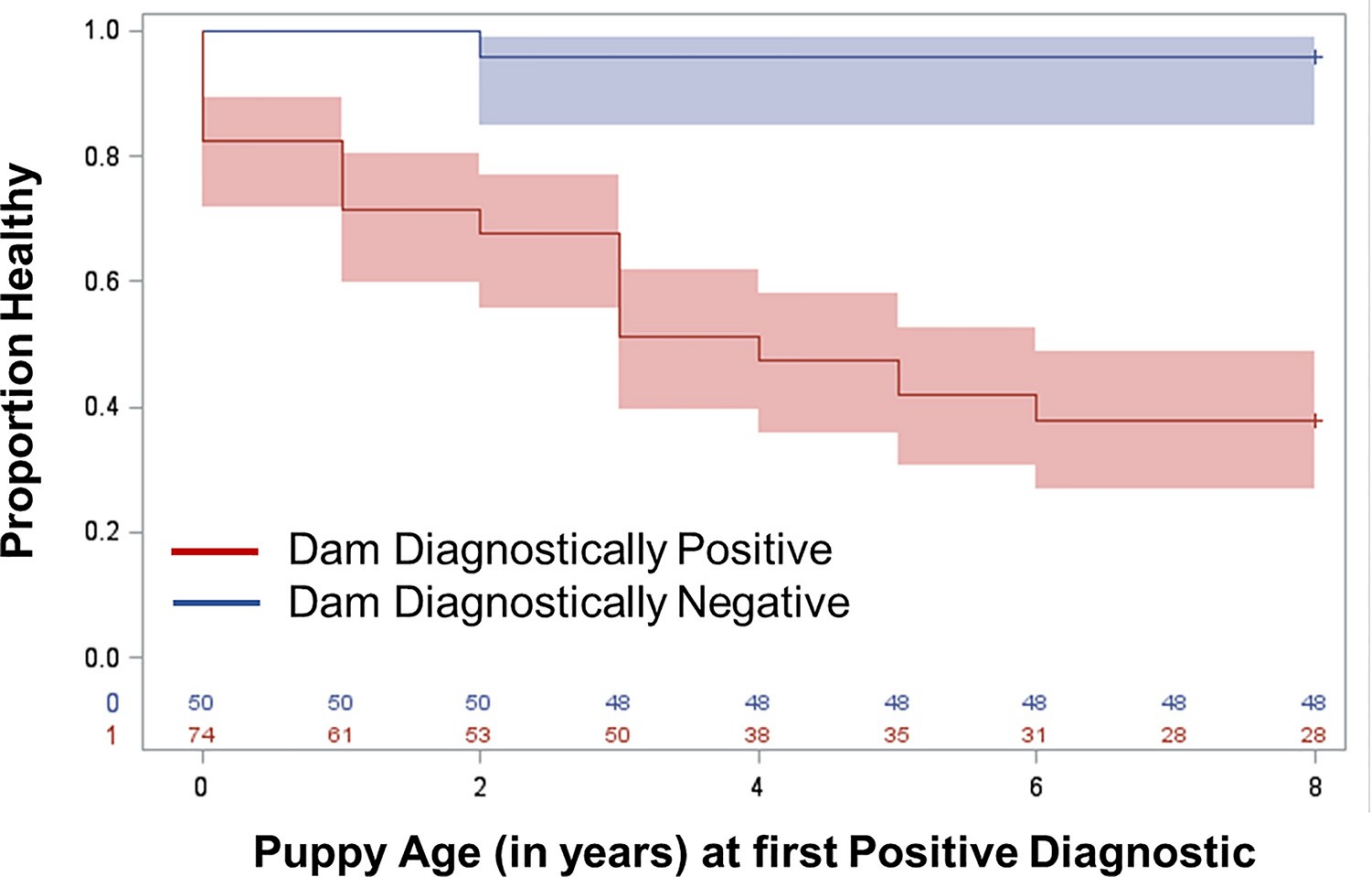
Kaplan-Meier time to offspring diagnostic positive based on dam’s diagnostic status ever. Proportion healthy refers to the proportion of dogs that were diagnostically negative via qPCR and ELISA and DPP CVL assay. Blue represents the diagnostic status of pups from dams who were diagnostically negative during their lifetime. Red represents the diagnostic status of pups from dams who were diagnostically positive at any point during their lifetime via qPCR or serology. Shaded area represents variance around the mean. (chi-squared: 26.28 p-value <0.0001).

Based on our findings via univariate and logistic regression, we were interested in evaluating the risk of becoming *Leishmania* diagnostic positive over years of a pup’s life based on it’s mother’s diagnostic status. To better assess when dogs became diagnsotically positive for *Leishmania*, time to event Kaplan-Meier curves were created. To visualize the overall relationship between age at which offspring became *Leishmania* diagnostically positive this was compared between the groups of dam *Leishmania* positve vs negative ever. Dogs born to positive dams (red) were statistically significantly more likely to become positive at a younger age than dogs born to negative dams (blue) (chi-square: 40.33, p-value <0.0001, **Fig 1**).

**Fig 2:**
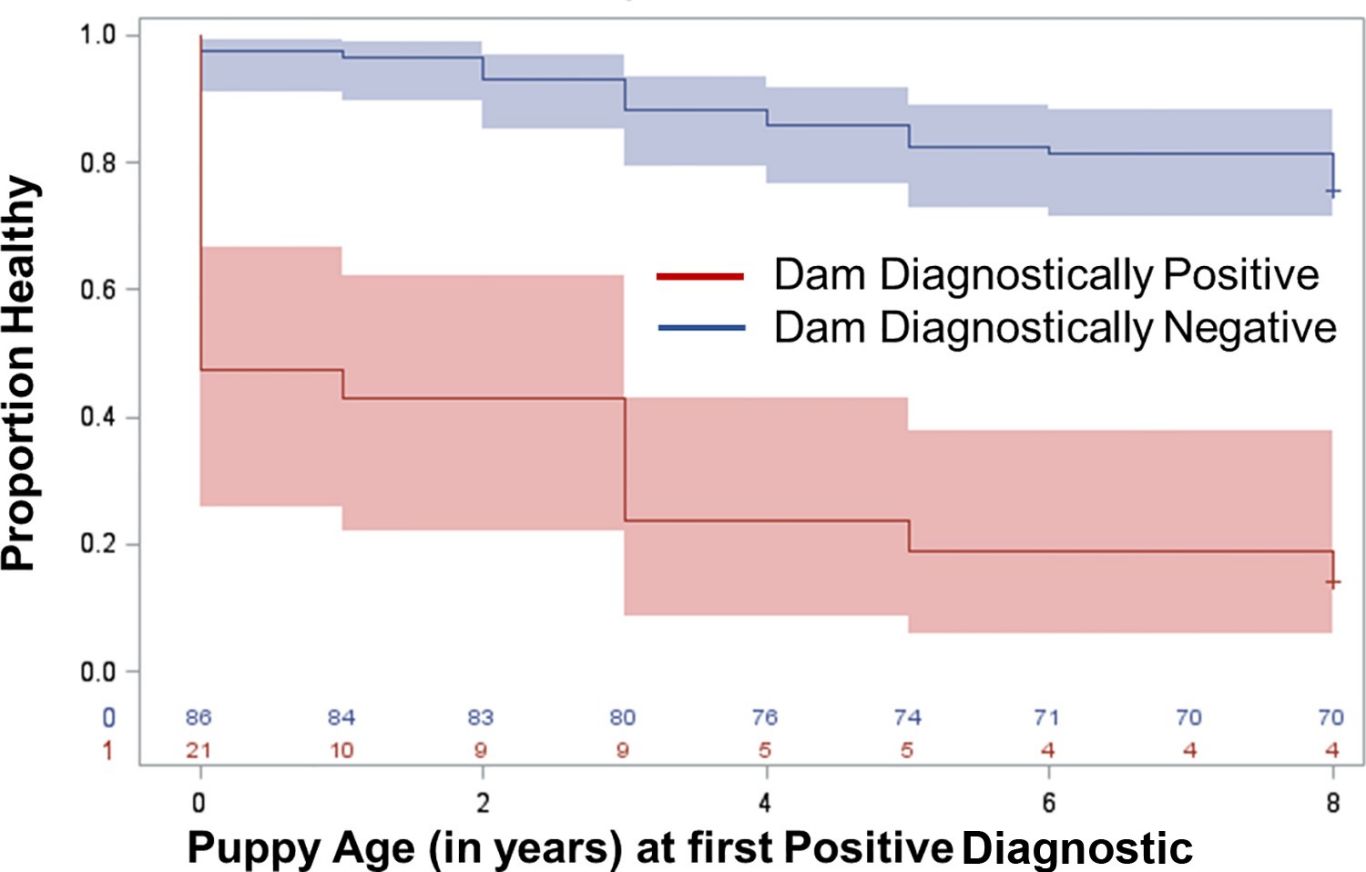
Kaplan-Meier time to dog diagnostic positive based on dam’s qPCR diagnostic status during the year of pregnancy. Proportion healthy refers to the proportion of dogs that are diagnostically negative via qPCR. Blue represents the diagnostic status of pups from dams who were diagnostically negative during year of pregnancy via qPCR. Red represents the diagnostic status of pups from dams who were diagnostically positive via qPCR during year of pregnancy. (chi-squared: 55.70 p-value <0.0001).

Based on the previous finding that dam qPCR status during the year she was preganant was also highly correlated with the pup becoming *Leishmania* diagnostic positive, dam’s qPCR status (negative during year of birth vs. positive) was utilized. Offspring born to dams who were qPCR positive (red) during the year they gave birth were significantly more likely to become positive for *Leishmania* via qPCR at younger ages than offspring from dams that were qPCR negative (blue) (**Fig 2**, chi-squared: 49.54 p-value <0.0001).

**Fig 3:**
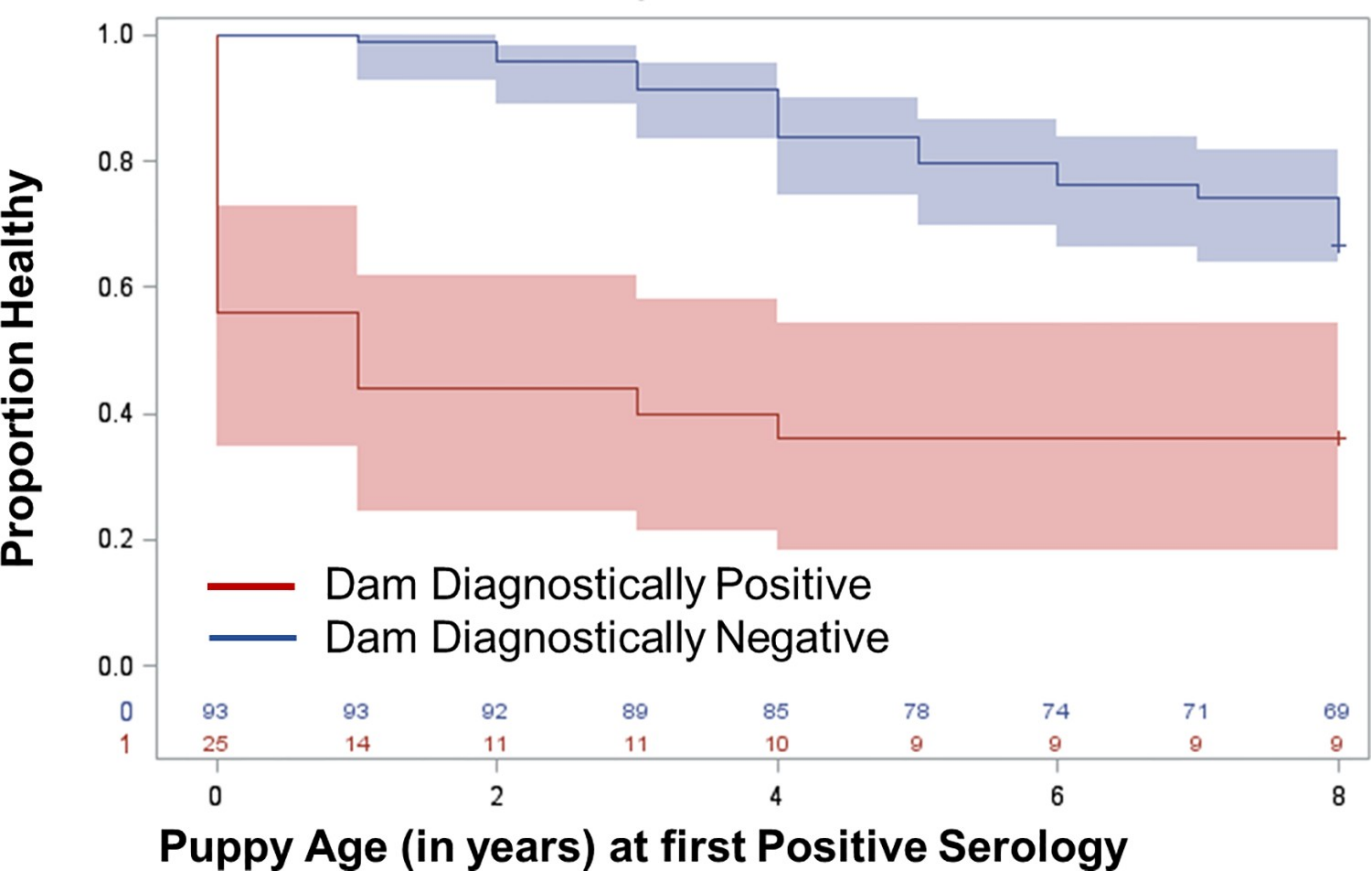
Kaplan-Meier time to dog diagnostic positive based on mother’s serological diagnostic status during pregnancy. Proportion healthy refers to the proportion of dogs that are diagnostically negative via ELISA and DPP CVL assay. Blue represents the diagnostic status of pups from dams who were diagnostically negative during year of pregnancy via ELISA and/or DPP CVL assay. Red represents the diagnostic status of pups from dams who were diagnostically positive via ELISA and/or DPP CVL during year of pregnancy.

This was similar to the relationship between dams who were seropositve during the year they gave birth and the age at which their puppies became seropositive for *Leishmania* (**Fig 3**, chi-squared 18.43, p-value <0.0001).

### Transmission of *L*. *infantum* persists across three generations of dogs

Within this study cohort we found two instances and three litters in which three generations of infected dogs were identified. In these specific families, on average the second generation had evidence of infection in 79.2% of dogs (seropositive or PCR positive at some point of their lives). To date, dogs in the third generation were 60.4% sero- or PCR positive for *L*. *infantum*. It should be noted that one of these litters are dogs currently 3 years old. These younger dogs may become qPCR or seropositive as they age and experience immunosuppressive conditions.

### Dogs diagnostically positive for *L*. *infantum* highly likely to die from clinical visceral leishmaniasis

A small subset of 20 dogs within the study were more closely followed through their entire lives and cause of death was established. Of the 20 dogs from infected dams for which a cause of death was identified 95%, or 19 of these dogs, died from clinical visceral leishmaniasis. The one dog identified as being diagnostically positive for *Leishmania infantum* but not dying from clinical visceral leishmaniasis died from a secondary infection with *Ehrlichia spp*. as identified at necrospy. Neither of the two dogs born to uninfected mothers found to be infected with *L*. *infantum* have died from VL, but this is a very small sample size.

### Basic reproductive number for vertical transmission of *Leishmania* above 4 new infections

The basic reproductive number for vertical transmission of *Leishmania* remains of interest in order to determine effectiveness of control efforts that are in many cases focused on vector transmission. R_0_ was calculated based on information regarding each litter from this population. On average, 64% of dogs born to a dam who were ever diagnostically positive for *Leishmania* will become positive in their lifetime. Using the average litter size of our population, between 6 and 7, we calculate an average R_0_ of 4.16.

## Discussion

A retrospective cohort study was performed to assess risk factors associated with vertical transmission of *Leishmania infantum* and a crude basic reproductive number was calculated for the population. The mother’s *L*. *infantum* diagnostic status during the year she was pregnant was a statistically significant risk factor for her offspring to be *L*. *infantum* positive during their lifetime, with a signficant 13 times greater risk of infection than dogs without maternal expsoure to *Leishmania*. Despite these dramatic findings in this retrospective cohort study, there is an overall paucity of reported cases of congential VL. This is likely for several reasons; first the diagnostic difficulties of confirming that a case is due to congential transmission vs. expsoure to sand fly transmitted disease in endemic areas. To date there is no way to distinguish *L*. *infantum* infection by route of transmission, so in endemic areas the presumption is that cases are vector borne, although this may not be true. The second reason is availability of treatment of mothers for ZVL during pregnancy reducing the maternal parasite load and therefore decreasing transmission to the child/offspring [27, 54]. This study is the first study to calculate the basic reproductive number and determine risk factors associated with vertical transmission of *Leishmania infantum* in a population where vertical transmission is the main route of transmission and there is no known vectorial transmission [55, 56].

Vertical transmission occurs not only in leishmaniosis but other infections as well, such as human immunodeficieny virus (HIV) and malaria [57, 58]. In HIV infection, anti-retroviral treatment during pregnancy and caesarean births have been associated with decreased risk of transmission likely due to a reduced exposure to the mother’s blood and virus [59]. In malaria, mothers with malaria during pregnancy are at risk of vertical transmission [60]. This is similar to CanL where dogs born to mothers that were qPCR positive during pregnancy had a much higher risk of becoming positive for *Leishmania*. This is likely due to the fact that a positive qPCR test identifies that there was parasite DNA in the blood which is shared between mother and pup across the placenta. The mother’s combined diagnostic status of seropositive or qPCR positive was a significant risk factor in predicting whether a puppy would become positive during their life. This was also reasonable as dogs become immunocompromised there can be increased disease progression and parasite replication with higher serological diagnostic values in dogs with more severe clinical disease [47, 50].

Within this study there were two sets of three generations of dogs that were followed and data indicating that transmission occurred across these generations. These results provide additional evidence that vertical transmission is capable of maintaining visceral leishmaniasis in a population over multiple generations.

Within this study two *Leishmania*-positive dogs were born to dams that were never qPCR or serologically positive for *Leishmania*. In the hunting dog community, dogs are commonly drafted or traded between groups and across international borders from endemic to non-endemic areas. Such movement of dogs greatly increases the difficulty of consistent testing across different locations and disease risk levels. This testing limitation may have led to a false negative status for the mother[61]. The two puppies that were identified as serologically/qPCR positive without maternal exposure could also have been exposed to *Leishmania* via fighting or wound cleaning of infected pen mates as blood to blood contact is possible due to the fact the dogs are housed in communal areas.

A small subset of 20 dogs (15% of the study population) were followed until death and a cause of death was identified. 100% of the dogs with an established cause of death were diagnostically positive for *Leishmania infantum* at some time throughout their life. Of those dogs with an established cause of death in this cohort, 95% died from clinical visceral leishmaniasis. These results highlight that without treatment many of these animals will progress with clinical disease. Therefore, it remains an important public health goal to identify ways to prevent *L*. *infantum* transmission from mother to child in both animals and people.

The basic reproductive number was calculated via an indivdual level model system, thus the number refers to the number of dogs in each litter that one mother could infect. This calculation provides a direct assessment of the R_0_ within this cohort. An R_0_ of approximately 4 (rounded to the nearest whole number to refer to number of puppies in the litter) shows that this disease is capable of maintaining at high levels within a population without vector transmission. The R_0_ of other diseases, such as influenza, which remain important public health concerns across the globe are as low as 2 [62]. Astonishingly, in comparison the R_0_ identified for an average canine litter coming from an infected dam was greater than 4, similar to the estimated basic reproductive number of smallpox [63]. As these studies all occur in an area where there is not holoendemic pressure of sand fly transmission, establishing the R_0_ and effect of vertical transmission in dogs from endemic areas would be valuable. These studies would all be limited by the inability to distinguish sand fly transmitted and vertical transmission once pups are born and it is hard to know the outcome of maternal infection on in utero pups.

Current control programs for leishmaniosis in countries where the disease remains endemic in both humans and animals include vector control, vaccination, and dog culling, which has been shown to be ineffective. Based on the data evaluated here, there is a significant need to also address vertical transmission through canine sterilization programs [64–66]. Recent studies have identified vaccination of infected/exposed asymptomatic dogs as safe, so vaccination to boost a better immunity prior to pregnancy may be of value to reduce transmission to the next generation [51]. Larger scale xenodiagnosis studies need to be performed to determine what skin burden of parasites is required to transmit CanL and the effectiveness of vaccination [67], allopurinol or additional (immuno)therapies to reduce parasite load immediately before or during pregnancy. Further analysis using Bayesian compartmental model techniques combining both vector and vertical transmission should be used to better understand the basic reproductive number for the full ecology of *Leishmania* infection in endemic areas and subsequently model how this number can be altered by public health control and prevention measures to assess elimination potential.

The findings of this study underscore the need for risk management through spaying and neutering animals by dog owners to reduce vertical transmision of *L*. *infantum* from their dogs. This action would decrease propagation of CanL within the canine reservoir for reduced transmission to people.
